# Potential role of gut bacteria in the development of hepatocellular carcinoma

**DOI:** 10.1099/acmi.0.000859.v6

**Published:** 2025-08-21

**Authors:** Bingqing Yan, Yuchao Wang, Ziqi Wang, Ailong Huang, Xiuxiu Jin, Tiantian Jiang, Hong Xue, Zheyu Shen, Shifang Wang, Haiyan Xu, Renfei Zhu

**Affiliations:** 1 Nantong Center for Disease Control and Prevention, Nantong, PR China; 2 Nantong Third People’s Hospital, Nantong, PR China

**Keywords:** gut bacteria, liver cancer, metagenome, pathway

## Abstract

Liver cancer is the fourth most deadly cancer, and early detection and timely treatment apparently play a crucial role in it. Intestinal bacteria affect the development of liver cancer through various pathways. In this study, the gut bacteria of liver cancer patients are analysed in detail by using metagenomic sequencing technology, and some of the bacterial species and metabolic pathways that may affect the development of liver cancer have been identified. Additionally, we identified bacterial factors that may impact key clinical indicators of the tumour. The findings of this study provide a scientific foundation for understanding the mechanisms underlying liver cancer development. This study freshened insights into clinical treatment strategies for liver cancer.

## Data Summary

The metagenomics sequencing data used in this study are accessible through the European Nucleotide Archive under the study accession number PRJEB75921.

## Introduction

Liver cancer is the fourth commonest cancer in the world [1]. The main risk factors for causing liver cancer are hepatitis B or C virus, alcohol abuse and non-alcoholic fatty liver disease [2], so early diagnosis is crucial for treatment and prognosis [3].

In recent years, the tumour microenvironment (TME) has been shown to play an important role in tumourigenesis and prognosis [4], and the gut microbiota usually regulates the immune tone of TME through immune modulation [5]. Gut-to-TME interactions, especially in non-gastrointestinal cancers, remain a critical area for discovery.

The gene set of the gut microbiome is estimated to be about 3 million genes, 150 times larger than the human genome [6], and the gut microbiota, including bacteria, fungi, archaea, protozoa and viruses, interact with the host to influence host physiology and health [7]. Yachida *et al*. reveal different stage-specific phenotypes of gut microbiota in colorectal cancer by metagenomic sequencing [8]. The tumour microbiome contributes to the aggressive phenotype of basal-like subtypes by characterizing the intratumour microbiota of different pancreatic adenocarcinoma subtypes using microbial metagenomic sequencing [9]. All these studies reveal the heterogeneity of cancers from a microbial standpoint.

The gut microbiota and liver cancer have complex interactions. An increasing number of studies have shown that the gut-liver axis influences the progression of liver diseases, such as liver inflammation, fibrosis, cirrhosis and cancer [10].

In addition, the microbiota interacts with the organism mainly through metabolites, and cholesterol metabolic homeostasis regulated by the gut microbiota is inextricably linked to hepatocellular carcinoma (HCC) [11]. The mevalonate pathway is a component of tumour initiation and progression [12]. However, the potential mechanisms of the gut microbiome in the development of HCC need to be deeply explored.

In this study, the gut bacteria of liver cancer patients were detected by metagenomic sequencing technology to search for the possible strains and mechanisms among them for the development of liver cancer and to explore the microbiota associated with some important factors and to find out the possible treatments by combining the clinical information.

## Method

### Sample source

A total of 20 human faecal specimens were collected, including 15 samples from liver cancer patients and 5 samples from normal individuals. The information of the samples is shown in Table 1.

**Table 1.** Sample quantity and information table

	Patients	Control
N	15	5
Male	8	2
Age	63.2±8.5	57.8±5.7

### Tool sample collection and DNA extraction

Bacterial DNA was extracted at Novogene Bioinformatics Technology (Beijing, China) using the SDS method from frozen faecal samples. DNA concentration and purity were assessed on 1% agarose gels, and DNA was subsequently diluted to 1 ng µl^−1^ using sterile water. DNA degradation degree and potential contamination were monitored on 1% agarose gels. DNA purity (OD260/OD280 and OD260/OD230) was determined using the NanoPhotometer^®^ spectrophotometer (IMPLEN, CA, USA). DNA concentration was measured using the Qubit^®^ dsDNA Assay Kit in Qubit^®^ 2.0 Fluorometer (Life Technologies, Carlsbad, CA, USA).

### Metagenomic shotgun sequencing

All samples were paired-end sequenced on an Illumina platform (insert size 350 bp, read length 151 bp, NovaSeq 6000) at Novogene Bioinformatics Technology (Beijing, China).

### Bioinformatics analysis

#### Data quality control

Adapter sequences were trimmed, and low-quality reads were filtered using fastp (version 0.23.4) [13]. Subsequently, host sequences were removed by aligning sequencing reads back to the host genome reference (hg38) with Bowtie2 (version 2.5.1) [14].

#### MetaPhlAn profiling

Taxonomic profiling of the metagenomic samples was performed using MetaPhlAn (version 4.0.6) [15], which utilizes clade-specific markers to provide pan-microbial (bacterial, archaeal, viral and eukaryotic) quantification at the species level. MetaPhlAn was run with parameters '--read_min_len 50 --force --no_map --add_viruses'. The relative abundance of microbial taxa in metagenomic samples is estimated based on the coverage of species-specific marker genes, as determined by MetaPhlAn.

### Functional analysis

A functional pathway table was generated with HUMAnN (version 3.6.1) [16] by employing the search mode of UniProt Reference Clusters (uniref90) and pathway MetaCyc. This table contains the functional potential of each metagenome sample, normalized using the relative abundance (relab) method. Subsequently, the normalized gene table was translated into KEGG Orthogroups, Pfam domains (Pfam), level-4 enzyme commission categories, EggNOG (including COGs) and carbohydrate-active enzyme, using the regrouped table script within HUMAnN.

### Alpha diversity

Unless otherwise stated, all statistical analyses were made in the R software, and *P* values<0.05 were considered statistically significant levels. Alpha diversity was assessed using Observed, Shannon and Pielou indices at the species level.

### Linear discriminant analysis effect size

Linear discriminant analysis effect size (LEfSe) analysis was implemented using the R package microeco [17], following the method proposed by Segata *et al.* [18] to identify microbial biomarkers significantly associated with group differences.

### Weight gene co-expression network analysis

Co-expression networks were constructed using the WGCNA R package [19]. Pairwise species correlations were computed using Spearman’s rank correlation coefficient, followed by power transformation to enforce scale-free topology.

## Results

### Sequencing results

Sequencing of 20 samples in this project yielded a total of 281.40 Gbp of raw data, and the final sequencing data (Clean Reads) used for analysis yielded a total of 275.77 Gbp, with a single-sample data size of 12–15 Gbp. The details are provided in the attached Table S1 (QC.xls).

### Diversity analysis

**Fig. 1.**
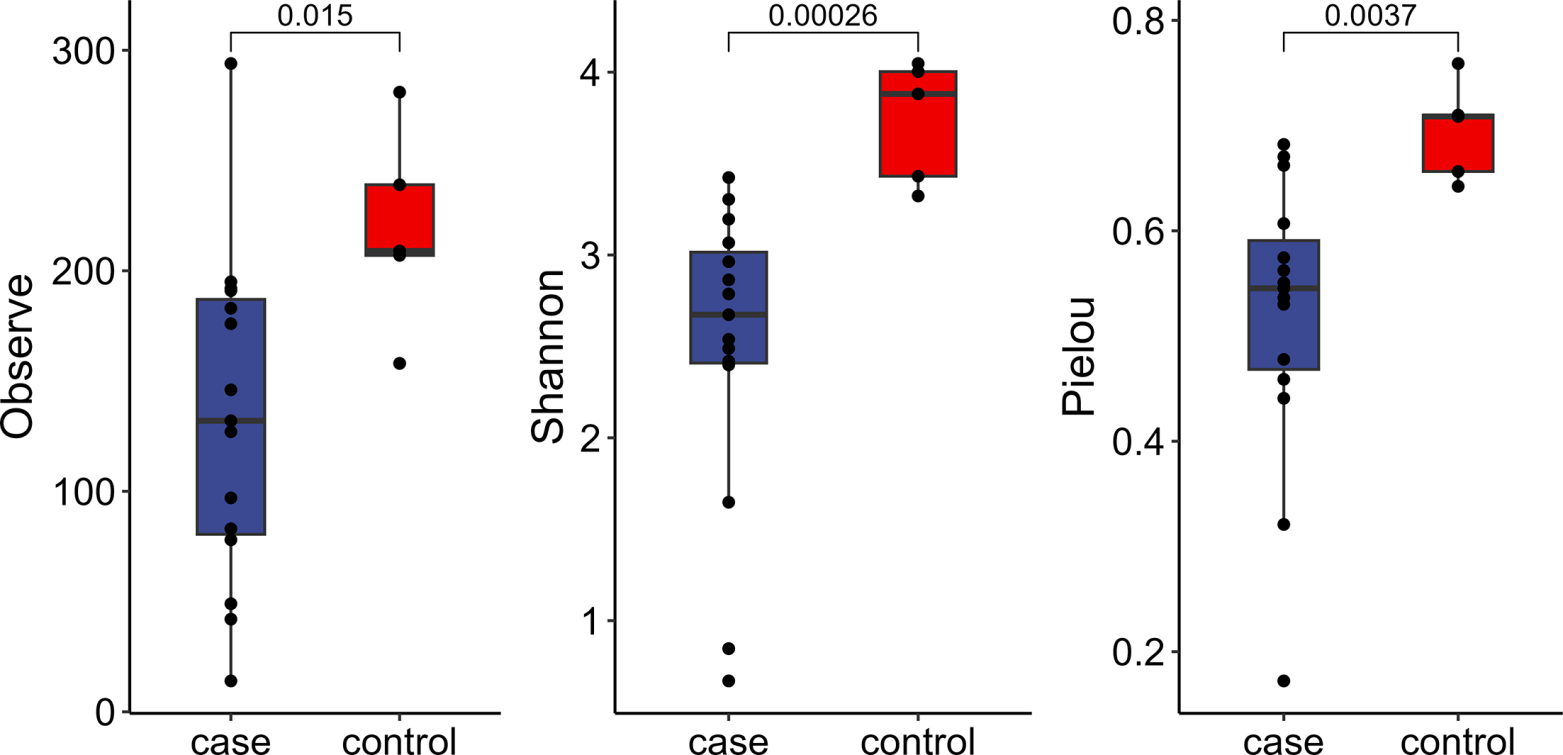
Results of *α*-diversity analysis at the species level.

At the species level, diversity analysis was performed on the HCC patient group and the normal control group (Fig. 1). The *α*-diversity indices (Observed, Pielou and Shannon) of the HCC patient group were all lower than those of the normal control group. This finding underscores a higher homogeneity within the gut microbiota of HCC patients, suggesting a disruption in the gut microbiota among individuals with liver cancer.

Boxes extend from the twenty-fifth to the seventy-fifth percentile, with the centre line indicating the median; the bottom whiskers indicate the minimum values, and the top whiskers indicate the seventy-fifth percentile plus 1.5-fold the inter-quartile distance (the distance between the twenty-fifth and seventy-fifth percentiles). *P* values between groups were determined using the Wilcoxon rank-sum test.

### Species composition in liver cancer patients

**Fig. 2.**
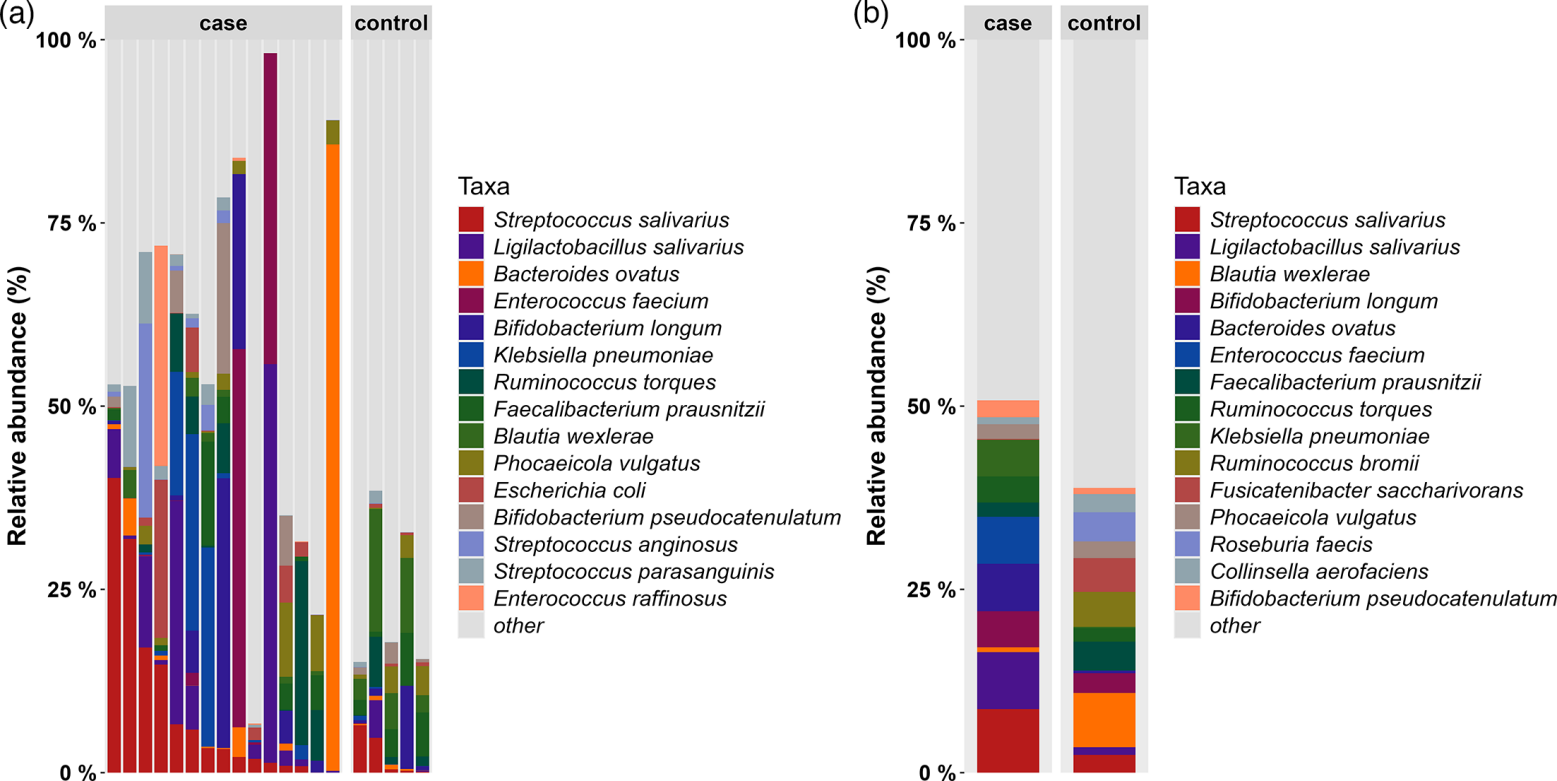
Histogram of the percentage of species composition. (**a**) Stacked bar plots showing the relative abundance of the top 15 bacterial species in individual faecal samples from HCC patients (*n*=15) and healthy controls (*n*=5); all remaining species are grouped as other. (**b**) Bar chart illustrating the mean cumulative relative abundance of the top 15 species in each group.

**Fig. 3.**
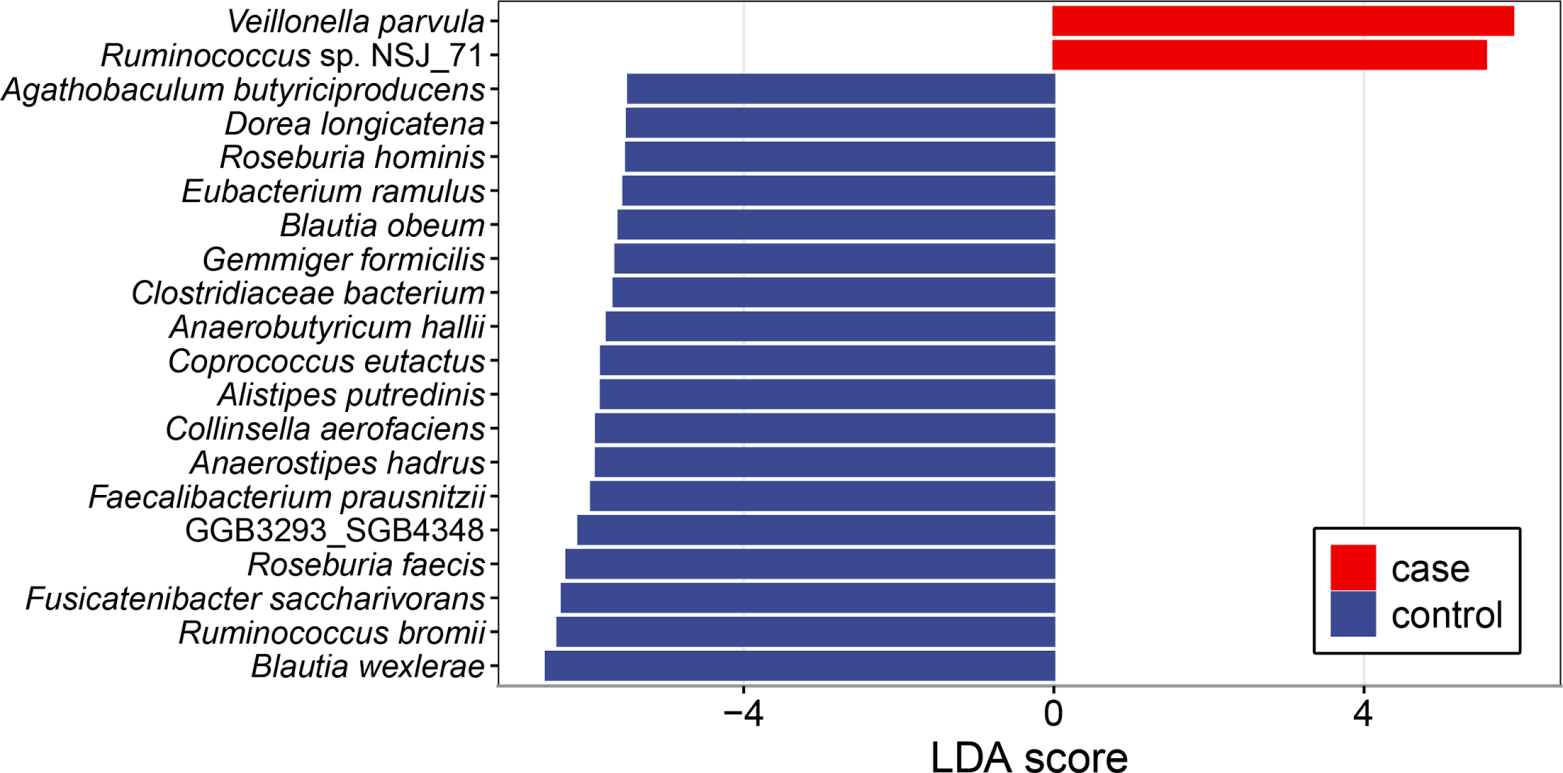
Schematic diagram of differential intestinal bacterial groups in HCC patients and normal subjects.

To examine the variations in gut microbiota among patients with HCC, the composition of the microbiota in the faecal samples was analysed (Fig. 2a), and it was found that there were large differences in the percentage of the various microbiota within the patient group and lesser differences within the normal group. It was found that *Streptococcus _salivarius* and *Ligilactobacillus_salivarius* were higher in the patients. To clarify the differences between patients and normal, LEfSe difference analysis was carried out on the subgroups to screen out the differentially abundant strains in HCC patients (Fig. 3, top 20 LDA species), and it can be found that highly abundant strains were *Veillonella_parvula* and *Ruminococcus_sp_NSJ_71*. The low abundance strains were more, including *Blautia_wexlerae*, *Fusicatenibacter_saccharivorans* and *Anaerostipes_hadrus*.

Linear discriminant analysis (LDA) was used to identify species with significant differences in relative abundance between HCC patients (*n*=15) and healthy controls (*n*=5). LDA scores reflect the magnitude of difference in relative abundance between groups.

### Sole hub species in liver cancer

**Fig. 4.**
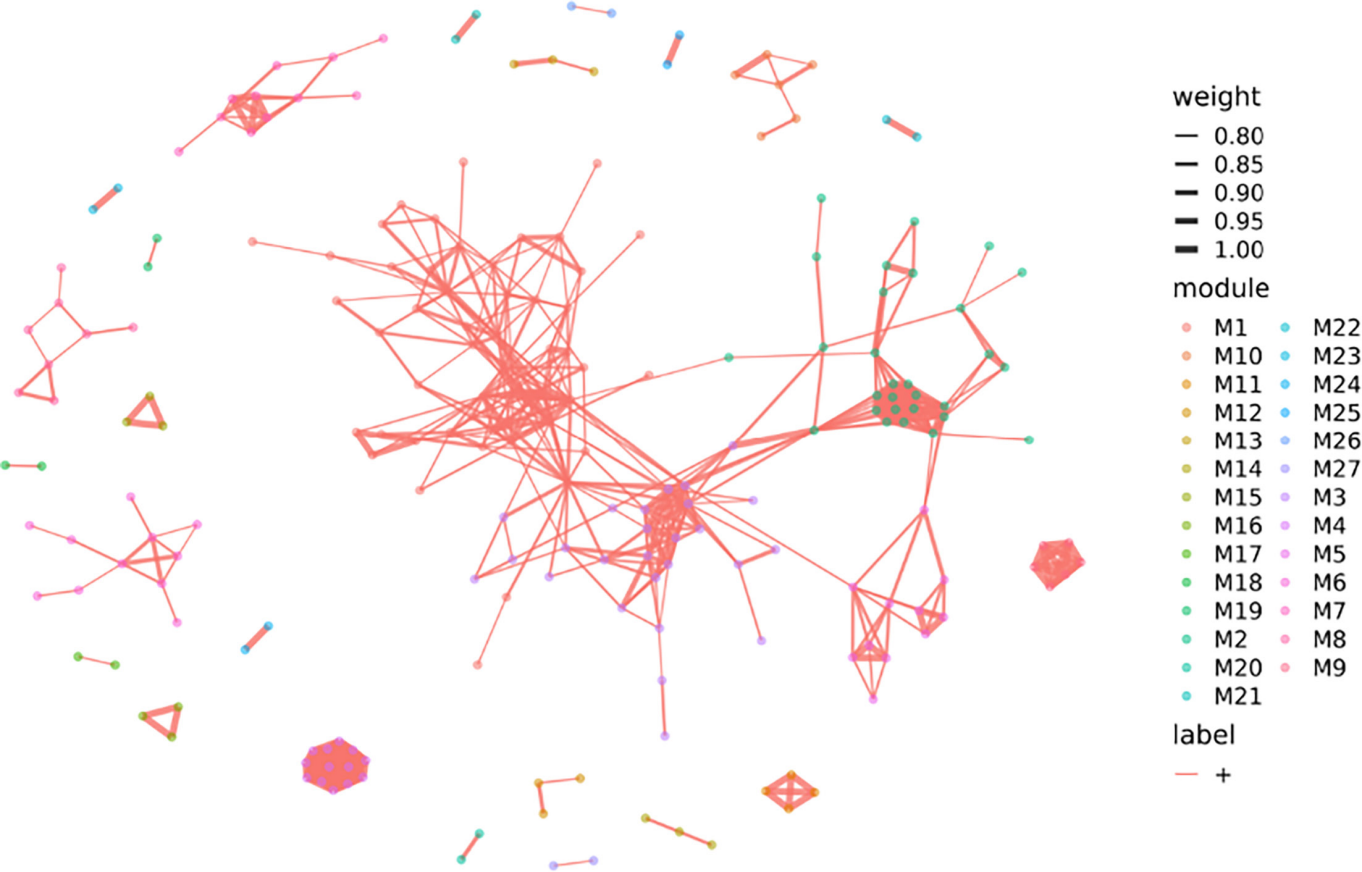
WGCNA map of the strain.

The WGCNA algorithm operates under the assumption that the network adheres to a scale-free distribution. In this distribution, various branches of the cluster dendrogram signify distinct microbial modules, characterized by high co-expression among microbes within each module and low co-expression among microbes belonging to different modules. A network diagram was drawn at the species level of the bacteria (Fig. 4), and the nodes of different colours in this network diagram represent the different bacteria, present in different modules. The connecting lines represent the correlation between modules, and the degree of thickness of the lines represents the strength of the correlation. Hierarchical clustering of topological overlap matrix-based dissimilarity values revealed 27 microbial modules, and the final network comprised 214 nodes, with *Roseburia hominis* identified as the sole hub species exhibiting marked depletion in HCC patients (*P*=0.0001).

WGCNA was used to construct a co-expression network of bacterial species. Nodes represent microbial species, coloured by module assignment. Edges indicate correlation strength between species. Modules were defined based on hierarchical clustering of topological overlap.

### Metabolic function analysis

**Fig. 5.**
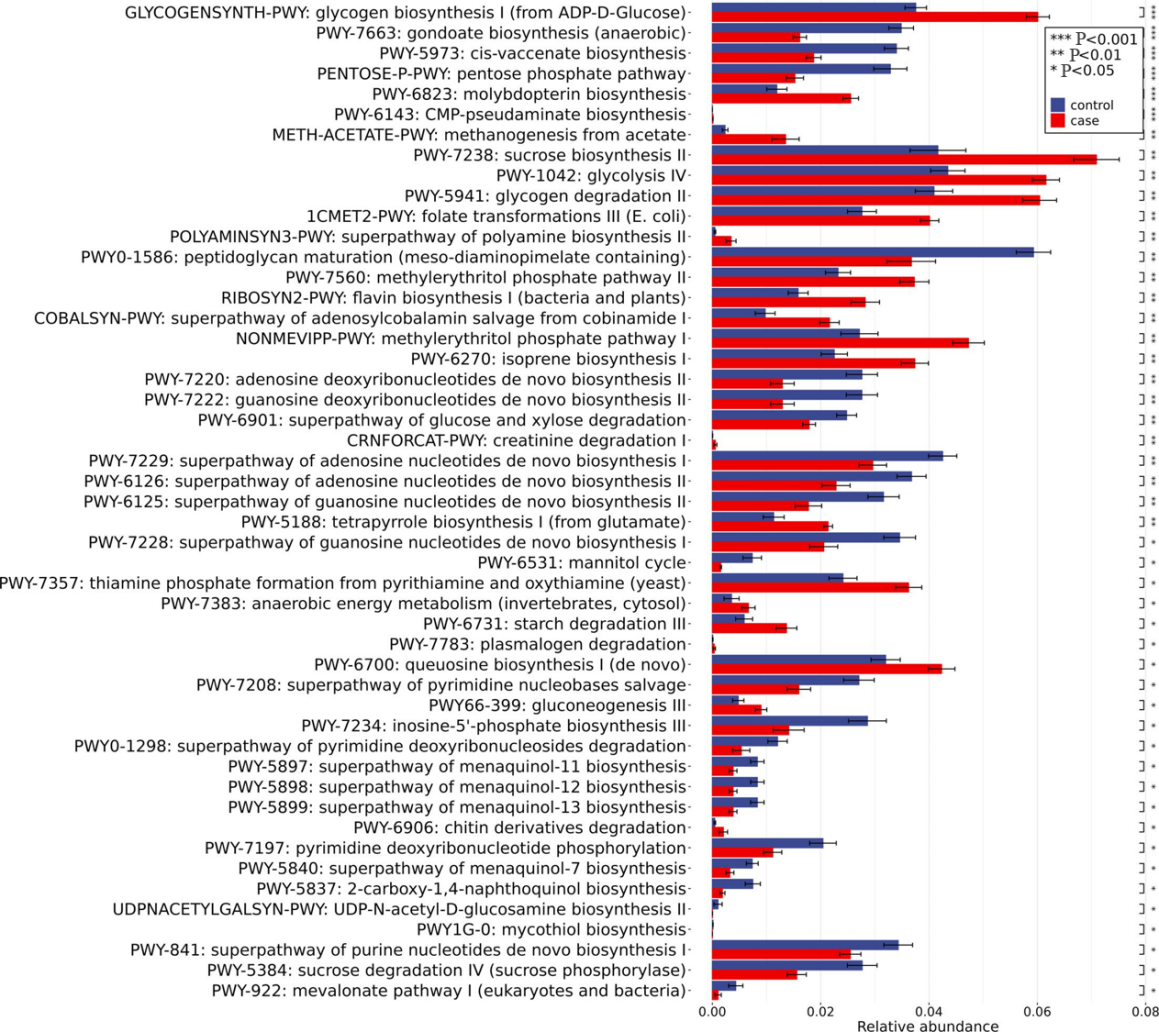
Metabolic function analysis diagram of differential bacteria groups.

Bacteria primarily influence the host through their metabolic products. To understand the effect of differential bacteria on the development of HCC, further metabolic function analysis of differential bacteria was carried out (Fig. 5). The highly expressed pathways mainly involved subpathways under the pentose phosphate pathway, such as the superpathway of pyrimidine nucleobases and the superpathway of purine nucleotides *de novo* biosynthesis, and the main relevant strain was *Veillonella_parvula*, which was of high abundance in HCC patients. The low expression pathway mainly involves the pathway of flavin biosynthesis, glycogen biosynthesis, etc., and the main related bacteria are *Blautia_wexlerae* and *Fusicatenibacter_saccharivorans*, which are low proportion in HCC patients.

Bar plots show all metabolic pathways with statistically significant differences in relative abundance between the two groups. Group comparisons were conducted using the Wilcoxon rank-sum test, and statistical significance is indicated as follows: * (*P*<0.05), ** (*P*<0.01) and *** (*P*<0.001).

### Correlation analysis of clinical indicators

**Fig. 6.**
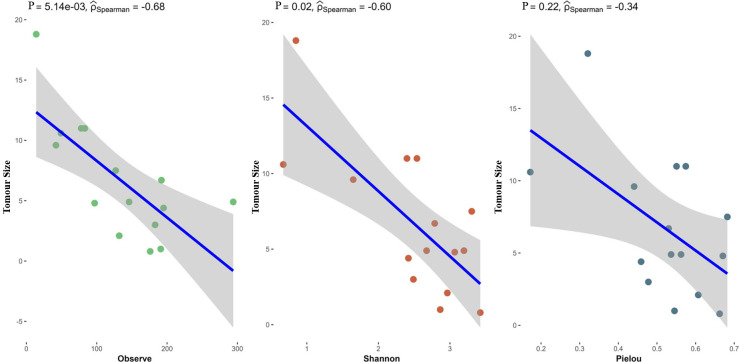
Correlation analysis between tumour size and alpha diversity of bacterial microbiota.

**Fig. 7.**
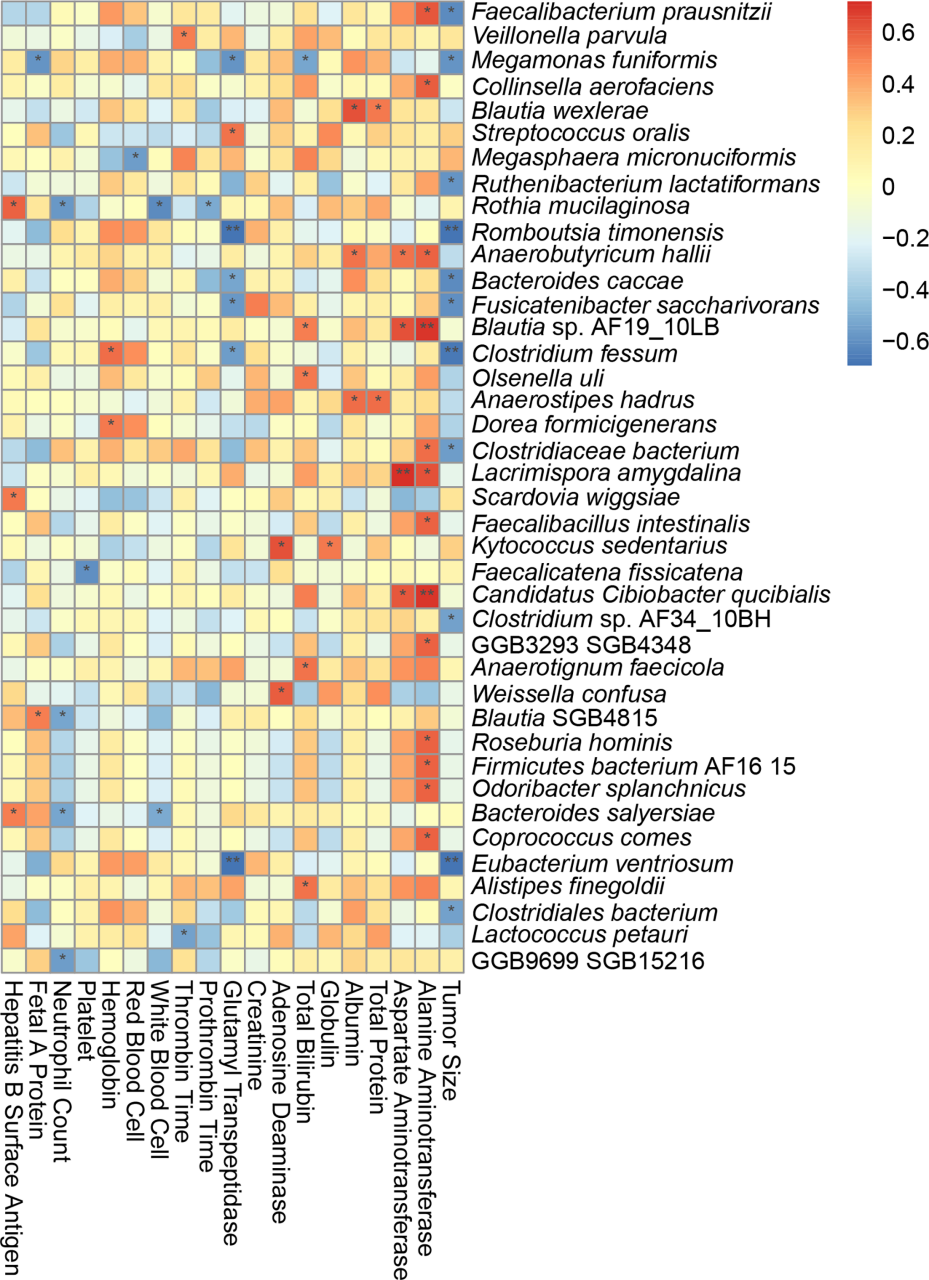
Heatmap of correlation between the differential strains and clinical indicators. Spearman correlation coefficients are displayed between differential bacterial species and clinical measurements. Significance levels are marked with * (*P*<0.05) and ** (*P*<0.01).

To delve deeper into the correlation between alterations in gut microbiota and HCC, this study gathered key clinical indicators of liver cancer and performed a correlation analysis with gut microbiota. The results demonstrated a trend distribution between tumour size, gut microbiota diversity and observational indices (*P*=0.00514, rho: −0.68), as well as a significant negative correlation between the Shannon index and tumour size (*P*=0.02, rho: −0.6). This indicates a notable negative correlation between differential microbiota abundance and tumour size (Fig. 6). Furthermore, this study conducted an in-depth analysis of the correlation between various bacterial populations and other clinical indicators (Fig. 7). The findings revealed that *Blautia wexlerae* exhibits a significant positive correlation with total protein and albumin, whereas *Fusicatenibacter_saccharivorans* displays a negative correlation with tumour size. These results suggest that bacteria may exist and play a role in phenotypic augmentation, potentially serving as a protective factor in cancer progression.

Scatter plots illustrating the associations between tumour size and alpha diversity indices (Observed, Shannon and Pielou), as assessed by Spearman correlation.

Spearman correlation coefficients are displayed between differential bacterial species and clinical measurements. Significance levels are marked with * (*P*<0.05) and ** (*P*<0.01).

## Discussion

The analysis of alpha diversity and species composition shows that the biodiversity of the bacterial microbiota in the faecal samples of patients with HCC is lower, and the distribution of the microbiota is more complex, which may be due to the use of antibiotics during the treatment of the patients.

Among the results of the analysis of variance, we can find a lower number of highly abundant strains and a significantly higher number of low-abundance strains with higher significance in patients with HCC using the LEfSe with the rank-sum test. The same results are presented in some studies [20]. Among the strains involved, such as *Blautia_wexlerae* and *Fusicatenibacter_saccharivorans*, both of which have reduced abundance in HCC patients, some studies have found that *Blautia *spp. and, especially, *B. wexlerae* are highly enriched as a symbiotic bacterium that is negatively associated with obesity [21], and obesity is also an important influencing factor in HCC. Stool samples from patients with cirrhosis show lower short-chain fatty acid (SCFA) levels and a corresponding reduction in the ability of batch fermentation to produce short-chain fatty acids, with butyrate production being the most abnormal. The higher the severity of liver disease, the more obvious these functional abnormalities are, and the abundance of *F. saccharivorans* was positively correlated with SCFA production [22], whose role in HCC development should not be ignored.

WGCNA results show that *Roseburia_hominis* is the only central node of the module, *R. hominis* is characterized by the production of short-chain fatty acids [23] and SCFAs can cross the blood-brain barrier and regulate the immune function of microglia [24], and in NAFLD-HCC, the gene function of the gut bacteria supports SCFA production. *In vitro* studies have confirmed that the composition of gut microbiota in NAFLD-HCC contributes to an immunosuppressive environment that is specific to HCC [25]. In particular, it has an important role in the glycogen biosynthesis pathway.

In this study, important clinical indicators of HCC patients are correlated with bacterial microbiota, which is of great significance for studying the abundance of microbiota in the occurrence and development of liver cancer. More and more evidence suggested that the gut microbiota plays a crucial role in the induction and progression of liver diseases [26]. However, there was no significant correlation between key factors such as Hepatitis B Virus (HBV) infection and bacterial microbial community diversity. The results of this study indicate a significant negative correlation between tumour size and the alpha diversity of bacterial microbiota, but the number of bacterial microbiota associated with HBV infection is relatively small. Some studies have revealed the relationship between HBV virus and tumour markers [27]. The results of this study suggest that the relationship between HBV virus and microbial community in liver cancer is more worthy of further exploration. There is a more complex association between microbial communities and liver cancer, which is not only reflected in the hepatic intestinal axis, but also in subsequent treatment. Given the limited sample size, the content revealed in this study is not yet comprehensive. We will continue to collect samples for further analysis.

## Supplementary material

10.1099/acmi.0.000859.v6 Uncited Table S1.

## Abbreviations

LDA: linear discriminant analysis
LEfSe: linear discriminant analysis effect size
TME: tumour microenvironment
